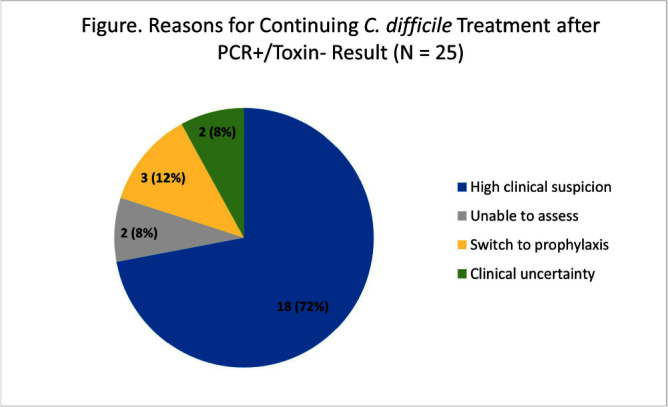# Clinician Interpretation and Management of Discordant PCR+/Toxin- Clostridioides difficile Testing Results Post-COVID

**DOI:** 10.1017/ash.2024.192

**Published:** 2024-09-16

**Authors:** Noah Boton, Preeti Mehrotra, Matthew Lee

**Affiliations:** Beth Israel Deaconess Medical Center

## Abstract

**Background:** Our institution utilizes a two-step algorithm consisting of an initial polymerase chain reaction (PCR) test with positive results reflexed to an enzyme immunoassay (EIA) for toxin. Institutional guidelines implemented during the COVID-19 pandemic recommended applying clinical judgment to patients with PCR+/Toxin- (discordant) results when determining if treatment is indicated. Pre-pandemic, we found that clinicians continued CDI-directed therapy in 56% of patients following the Toxin- result. Our study aims to identify how clinicians interpret PCR+/Toxin- results and reasons for management decisions post-pandemic. **Methods:** At an academic medical center, we conducted a retrospective cohort study of the first 50 inpatient charts with PCR+/Toxin- results from August to October 2022. Data was abstracted from clinical, pharmacy, and microbiology databases. The primary outcome was the proportion of patients continued on CDI-directed therapy for ≥24 hours after the Toxin- result became available. Secondary outcomes included the proportion of patients prescribed a full treatment course for CDI and the reasons for continuing treatment. **Results:** There were 37 patients (74%) who started CDI-directed treatment after initial PCR+ **Results:** Of these patients, 59% (22/37) were continued on treatment for ≥24 hours after the Toxin- result (primary outcome). 77% (17/22) of these patients who met the primary outcome completed full treatment courses. Three patients were transitioned to prophylaxis dosing after the Toxin- result. The most common reason for continuing treatment after discordant results was high clinical suspicion for CDI (Figure). There were no CDI-related complications in this 50-patient cohort. In immunocompromised patients, 70% (16/23) started treatment after initial PCR+ results and 81% (13/16) met the primary outcome. In patients admitted specifically to immunocompromised inpatient services, 90% (9/10) started treatment after initial PCR+ results and 100% (9/9) met the primary outcome. **Conclusion:** The majority of patients started on treatment after the PCR+ result were continued on treatment following the Toxin- result, though several of these patients did not complete full treatment courses. Treatment rates were similar to our pre-pandemic baseline. When patients were continued on treatment after discordant results, clinicians cited appropriate high clinical suspicion. Notably, every patient admitted to an immunocompromised service was continued on treatment after Toxin- **Results:** Overall, clinicians are following institutional guidelines by applying clinical judgement when interpreting discordant **Results:** Further research will help identify what variables affect clinician interpretation and management practices for discordant results, which will help shape institutional guideline updates, clinician education, and additional stewardship interventions.

**Figure:**